# Identifying Genes Relevant to Specific Biological Conditions in Time Course Microarray Experiments

**DOI:** 10.1371/journal.pone.0076561

**Published:** 2013-10-11

**Authors:** Nitesh Kumar Singh, Dirk Repsilber, Volkmar Liebscher, Leila Taher, Georg Fuellen

**Affiliations:** 1 Institute for Biostatistics and Informatics in Medicine and Ageing Research, Department of Medicine, University of Rostock, Rostock, Germany; 2 Institute for Genetics and Biometry, Leibniz Institute for Farm Animal Biology, Dummerstorf, Germany; 3 Institute for Mathematics and Informatics, Ernst Moritz Arndt University of Greifswald, Greifswald, Germany; University of Westminster, United Kingdom

## Abstract

Microarrays have been useful in understanding various biological processes by allowing the simultaneous study of the expression of thousands of genes. However, the analysis of microarray data is a challenging task. One of the key problems in microarray analysis is the classification of unknown expression profiles. Specifically, the often large number of non-informative genes on the microarray adversely affects the performance and efficiency of classification algorithms. Furthermore, the skewed ratio of sample to variable poses a risk of overfitting. Thus, in this context, feature selection methods become crucial to select relevant genes and, hence, improve classification accuracy. In this study, we investigated feature selection methods based on gene expression profiles and protein interactions. We found that in our setup, the addition of protein interaction information did not contribute to any significant improvement of the classification results. Furthermore, we developed a novel feature selection method that relies exclusively on observed gene expression changes in microarray experiments, which we call “relative Signal-to-Noise ratio” (rSNR). More precisely, the rSNR ranks genes based on their specificity to an experimental condition, by comparing intrinsic variation, i.e. variation in gene expression within an experimental condition, with extrinsic variation, i.e. variation in gene expression across experimental conditions. Genes with low variation within an experimental condition of interest and high variation across experimental conditions are ranked higher, and help in improving classification accuracy. We compared different feature selection methods on two time-series microarray datasets and one static microarray dataset. We found that the rSNR performed generally better than the other methods.

## Introduction

DNA microarrays can be classified into static experiments, where a snapshot of gene expression in different samples is measured, and time series experiments, where a temporal process is measured over a period. While static experiments may reveal genes that are expressed under specific conditions, time series experiments may help in determining the temporal profiles of the genes expressed under a specific condition, as well as interactions between them [1].

An interesting problem in microarray analysis is the classification of unknown expression profiles with the goal of assigning them to one or many predefined classes. Such classes represent various phenotypes, for example, diseases. Moreover, classifying microarray data by cross-comparing microarray data from different laboratories and phenotypes could be helpful not only to identify unknown samples, but to reveal obscure associations between complex phenotypes, such as shared pathogenic pathways among different diseases. Such approaches have been made more feasible in recent years with the availability of large database repositories of high throughput gene expression data, such as the Gene Expression Omnibus (GEO) [2]. However, classifying microarray data is a challenging task, mainly because of the large number of non-informative variables involved: a regular microarray dataset comprises from 6000 to 60,000 genes [3]. First, as for any large-scale dataset, classification algorithms require substantial computational resources. In this regard, the current affordability of massive computer power and recent advent of cloud computing have opened new possibilities. In particular, web-based workbenches such as Galaxy [4], standalone, comprehensive collections of data analysis and integration tools such as Chipster [5], and large, active communities devoted to open source and open development projects such as the Bioconductor [6] have made data-intensive biology available to virtually all scientists. Second, and more importantly, the performance of most classification algorithms is affected by the relatively low signal-to-noise ratio of such datasets. Furthermore, because often only a few dozens of samples are available, most algorithms face the risk of overfitting [7]. Reducing the number of genes using feature selection methods not only results in a more efficient management of the computational resources and a lower the risk of overfitting, but also enables a better biological understanding of the data.

Many studies have shown that integrating microarray data with additional biological information improves classification accuracy. For example, Bar-Joseph et al. discuss how protein-DNA binding data and protein interaction data can be used to constrain the number of hypotheses that can explain a specific expression pattern [1]. More generally, protein interactions and their dynamics are considered helpful, and even essential for understanding biological processes [8]. For instance, de la Fuente argues for the importance of integrating gene expression data with network information to identify dysfunctional regulatory networks in disease [9], and, consistently, several studies suggest that pathway dysregulation is a stronger biomarker for cancer compared to the dysregulation of individual genes [10]–[12]. Also, Ma et al. showed that an approach to identifying genes associated with a given phenotype that combines expression and protein interaction data outperforms other approaches that use either gene expression or protein interaction [13]. Similarly, Wu et al. used both gene expression and protein interaction data to prioritize potential cancer-related genes for further investigation, with encouraging results [14]. Additionally, gene combinations have been shown to be more effective than individual genes in classifying cancer *versus* healthy samples [15], and pluripotent *versus* non-pluripotent cells [16]. Consequently, although the exact contribution of protein interaction information is difficult to assess [17], such information is potentially useful to identify features with biological relevance to specific experimental conditions, and improve microarray classification.

Signal-to-noise (SNR) ratios have been extensively used in various fields. In image processing, the SNR is defined as the mean of the variable being measured divided by its standard deviation [18]:
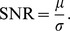



In this case, the standard deviation represents noise and other interference in comparison to the mean. The reciprocal of the SNR is known as coefficient of variation (CV), which has been widely applied as quality control and validation method for the analysis of microarray assays, see, e.g., [19]–[21]. For instance, Raman et al. used the CV to investigate differences in the variability of expression levels with regards to quality control, and found that the CV was greater for the microarrays that failed the quality control inspection compared to those that did not [21]. The SNR has also been proposed and successfully employed as a feature selection method for classification problems. For the sake of clarity, we will refer to the Signal-to-Noise ratio used for classification problems as 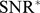. In the context of feature selection for microarray classification, the 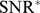 measures the effectiveness of a feature in discriminating between two classes and is defined as [22]–[26]:
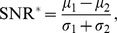
where, *μ_1_* and *μ_2_* are the mean expression value for class 1 and class 2 respectively while *σ_1_* and *σ_2_* are the standard deviation for class 1 and class 2 respectively.

The 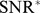 has been extensively used with some modifications or in combination with other methods. For example, Lakshmi and Mukherjee used a “maximized” 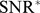 that identifies features with the aim of increasing the distance between the category profiles [27]; Mishra and Sahu used 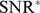 in combination with various clustering methods and classifiers [28]; and Goh et al. applied multiple passes of feature selection using 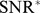 and Pearson correlation coefficient [29].

Here, we show that identifying biologically relevant features substantially improves microarray classification. First, we explore the addition of protein interaction information as a means to select features specific to particular experimental conditions and improve microarray classification. We found that, in the form presented here, the addition of protein interaction information resulted in no improvement in classification. Second, we introduce a novel feature selection method based on the SNR, which we call the “relative signal-to-noise ratio” (rSNR). Given a microarray dataset comprising various experimental conditions, the rSNR is a feature ranking method that ranks genes based on their specificity to a given experimental condition, by comparing variation in gene expression within that particular experimental condition, with variation in gene expression across other experimental conditions. Basically, the rSNR can be expressed as a quotient of SNRs or CVs, and in practice, gives higher rank to genes with high expression values and low standard deviations in the experimental condition of interest. We tested this and other feature selection methods on two time-series microarray datasets and one static microarray dataset. The rSNR method performed generally better than other feature selection methods, and its application substantially improved classification accuracy. Our results also suggest that the rSNR rank could be used to reduce the number of genes representing a microarray experiment in a database, hence, making searches across the entire database more efficient.

## Methods

### Classifying Gene Expression Time-course Data Using Correlation Tests

To classify time course microarray experiments we adopted a nearest neighbor approach based on Pearson correlation. Training and test data consisted of gene expression profiles from two or more different time points. When considering test datasets comprising more than two time points, we first split the data into test subsets consisting of pairs of consecutive time points. We then evaluated the classification on each of these subsets, and decided the final classification of the entire test data by majority voting.

With the goal of improving classification performance we examined different data features. First, we evaluated different manners in which gene expression profiles from two different time points can be combined into what we call transition profiles. Then, we incorporated protein interaction information from the STRING database into the definition of such transition profiles. Finally, we compared different feature selection approaches to extract the genes that are the most relevant to specific biological conditions.

### Notation

In the following, we adopt notation by Hafemeister et al. [30]. Let

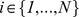
 represent the experimental conditions/queries,

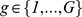
 be the running index of the genes,

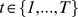
 denote the time points,

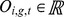
: expression value of gene *g* of experiment *i* at time *t*,

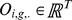
: expression time course of gene *g* of experiment *i*,

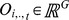
: expression value of all genes at time *t* of experiment *i*. This vector of expression values is also called the expression profile at time *t* of experiment *i*,

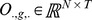
: collection of all expression values of gene *g* for all experiments *i* across all time points *t*,

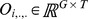
: collection of all expression values for experiment *i* for all genes *g* across all time points *t*.

### Datasets

We trained and tested our method on the two time-series microarray datasets used by Hafemeister et al. [30], consisting of expression data describing *Arabidopsis thaliana* stress response (“AtGenExpress”) [31] and the EDGE toxicology database (“EDGE”) [32]. We further tested our method on a static microarray dataset used by Engreitz et al. [33]. A brief description of each of the three datasets follows.

#### AtGenExpress

We downloaded this dataset from the TAIR database [http://www.arabidopsis.org/, [31]]. It consists of microarray expression data from *Arabidopsis thaliana* when exposed to various stress treatments. The dataset has 232 samples comprising 9 stress treatments and 2 tissue types (root and shoot) at 8 time points (*T* = 8), with 2 replicates at each time point. In total, there are 18 unique combinations of stress treatments and tissue types to which we will refer as experimental conditions (*N* = 18). We will refer to the variables in the experiment, i.e., stress treatment and tissue type, as “experimental factors”. The original dataset contained expression values for 22810 genes. Hafemeister et al. reduced it to 2074 genes by applying a 2-fold-change filter [30]. All our analyses are based on the reduced dataset of 2074 genes (*G* = 2074).

#### EDGE

We obtained this dataset directly from its original author [32]. It contains gene expression values from mice when treated with various toxins at different dosage levels. The dataset has 216 samples comprising 7 toxin treatments at various dosage levels at up to 12 time points, ranging from 2 to 192 hours (*T* = 12), with replicates ranging from 1 to 40 at different time points. In total, there are 11 unique combinations of toxin treatments and dosage levels, to which we will refer as experimental conditions. We will refer to the variables in the experiment, i.e., toxin treatment and dosage level, as “experimental factors”. The dataset contains expression values for 1600 genes (*G* = 1600). Since our validation framework requires samples from at least 4 time points, we discarded experimental conditions with fewer than 4 time points. This resulted in a reduced EDGE dataset with 6 unique experimental conditions (*N* = 6). All our analyses are based on this reduced dataset.

#### Engreitz dataset

We obtained this dataset from Jesse M. Engreitz [33]. It is a collection of 32 disease-associated microarray experiments. These microarray experiments compared normal to diseased tissue for Duchenne muscular dystrophy, breast cancer and Huntington’s disease. Hence, these 32 microarray experiments can be categorized into 3 experimental conditions (*N* = 3), based on the associated diseases. Each microarray experiment consists of arrays or expression profiles which can be categorized in either “normal” or “diseased” (*T* = 2). Each microarray experiment consisted of a unique set of genes. In order to create a single dataset with around 3000 common genes, we excluded 5 microarray experiments. The reduced dataset contains 3378 genes (*G* = 3378). The expression values in the reduced dataset were quantile normalized.

### STRING Database

We obtained protein interaction information from STRING, a database of known and predicted protein-protein (and protein-gene) interactions [http://string-db.org/, [34]–[39]]. The interactions in STRING are derived from various sources and contain both physical and functional associations. Each interaction in STRING is associated with a confidence score, which is associated with the probability that the interaction exists.

### Gene Expression and Transition Profiles

As test/training data we used pairs of expression profiles from two time points of the same experimental condition. We converted each of these pairs of expression profiles into a vector of expression values to which we refer as *transition profile*. We used two different kinds of transition profiles:

- **Differential transition profile (DTP).** The DTP measures the change in expression value for all genes between two time points *t_x_* and *t_y_*. The DTP of an experimental condition *i* for a pair of time points *t_x_* and *t_y_* is calculated as:

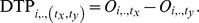
where, 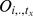 and 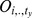 are vectors containing the expression values of all genes under experimental condition *i* at time points *t_x_* and *t_y_* respectively.

- **Mean transition profile (MTP).** The mean transition profile is the mean expression value for all genes between two time points. The MTP of an experimental condition *i* for a pair of time points *t_x_* and *t_y_* is calculated as follows:

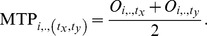
where, 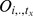 and 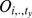 are defined as for the DTP.

To obtain a transition profile, two expression profiles are combined into a single vector of expression values. Hence, we also compared transition profiles with single gene expression profiles:

- **Time point expression profile.** Here, we based the classification on individual gene expression profiles. To make the comparison with the classification based on pairs of gene expression profiles fair, the individual gene expression profiles were taken from a set of two profiles (see subsection below).

We evaluated the performance of our method using the above-mentioned expression and transition profiles on the AtGenExpress and EDGE datasets.

### Similarity Between Expression and Transition Profiles

We classified gene expression and transition profiles according to the 1-nearest neighbor rule (1NN). Similarity between expression or transition profiles was evaluated using the Pearson correlation coefficient. Thus, for a given transition profile selected as test data, we computed, pairwise, the Pearson correlation coefficient between it and all the transition profiles in the training data. Then, we examined the Pearson correlation coefficient of each pair, and labeled the test data with the experimental condition of the transition profile in the training data for which we obtained the highest Pearson correlation coefficient. To make the comparison between expression and transition profiles fair, in the case of time point expression profiles, the test data consisted of two expression profiles. We computed, pairwise, the Pearson correlation coefficient between all the expression profiles in the test data and all the expression profiles in the training data. Finally, the test data were labeled with the experimental condition of the expression profile in the training data for which we obtained the highest Pearson correlation coefficient.

### Leave-one-out Cross-validation

We evaluated the predictive power of our method for different choices of parameters and features using leave-one-out cross-validation. In the following, we assume that pairs of time-points are sorted by time in ascending order.

- **AtGenExpress and EDGE datasets.** The test dataset was generated by randomly selecting 2 time points from a given experimental condition. For these 2 time points, we used one of the replicates as test dataset, and excluded all other replicates from the training and test datasets. The training dataset consisted of all the remaining time points for this experimental condition together with all the time points for other experimental conditions. We generated ten such test/training datasets for each experimental condition, and repeated this random sub-sampling validation procedure 30 times for AtGenExpress, and 100 times for EDGE.
- **Engreitz dataset.** This dataset contains 27 microarray experiments categorized into 3 experimental conditions. Each microarray experiment has expression profiles in “normal” and “diseased” states. We treated “normal” as time point 0 and “diseased” as time point 1. For the cross validation, we selected one microarray experiment (2 time points) as test data, and the rest as training data. This process was repeated so that each microarray experiment was selected as test data exactly once.

### Evaluation of the Classification

If the predicted experimental condition for the test data is the same experimental condition from which the test data had been taken, then, the classification is correct; otherwise, the classification is incorrect. We used the accuracy to evaluate the classification results for different methods, parameters, and features:
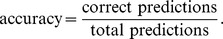



For the Engreitz dataset, in addition to the accuracy we used the area under the Receiver Operating Characteristic (ROC) curve (AUC). Each of the 27 microarray experiments were used to query the remaining 26 microarray experiments exactly once in order to determine whether they correspond to the same experimental condition (i.e., disease). Thus, given a query microarray experiment, we computed 26 correlation coefficients. Then, we defined a cut-off on those correlation coefficients. All microarray experiments for which we obtained correlation coefficients higher than the cut-off were classified as “positives”. Out of these positive microarray experiments, those that indeed corresponded to the experimental condition of the query microarray experiment were considered “true positives” (TP); those corresponding to a different experimental condition were considered “false positives” (FP). We then computed the true positive rate (TPR) and false positive rate (FPR). The ROC curve represents the TPR as a function of the FPR for different cut-off values. The AUC reported is the average computed for all 27 microarray experiments taken as query.

### Generation of Linked Gene and Link Dataset

We also investigated the effect of adding protein interaction information on microarray classification. We retrieved this information from the protein interaction database STRING [http://string-db.org/ [34]–[39]], and refer hereafter to interactions (both omnidirectional and directional) obtained from this source as “links”. First, we created a dataset of genes for which we observe at least one interaction in STRING. We decided on these interactions using a range of STRING confidence score thresholds. We call this dataset “linked gene dataset”. Second, we converted the “linked gene dataset”, into a dataset describing interactions, rather than genes. We call this dataset “link dataset”. Link datasets include exactly one link for each pair of genes in the “linked gene dataset” for which there exists an interaction in STRING. Then, similarly to Warsow et al. [40], we define the “expression value” of a link as the mean expression value of the two genes involved:
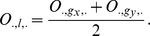
where link *l* implies a protein interaction between gene *g_x_* and *g_y_*. and *O_.,l,._* is a *N*×*T* matrix containing the link expression values of the link *l* for all experiments and time points.

We compared the classification performance of classifiers relying on gene, linked gene and link datasets.

### Feature Selection Methods

Microarray experiments are intrinsically noisy in that they involve a very large number of genes, most of which exhibit irrelevant variation [41]. In this context, the aim of feature selection methods is to identify and exclude such non-informative genes. We evaluated different feature selection methods. First, we used information from STRING to select biologically relevant genes, and created a linked gene dataset and a link dataset consisting of only these selected genes. Second, we developed the rSNR, a novel feature selection method that identifies biologically relevant genes based on their SNR. Finally, we compared our classification results with those obtained by selecting genes randomly. Except for the rSNR, which is explained in detail in the following section, the remaining aforementioned methods are outlined as follows:

1. **STRING-based link selection.** Our link datasets included only those links representing interactions between genes present on the microarray and with a confidence score in STRING greater than a given cut-off, which was selected from {0, 250, 500, 750, 900}.
2. **STRING-based gene selection.** Our linked genes datasets included only those genes present on the microarray and for which there is an interaction in STRING with a confidence score greater than a given cut-off. As for the STRING-based link selection, cut-off scores were selected from {0, 250, 500, 750, 900}. It follows that for each cut-off score, the link and linked genes datasets included information of exactly the same genes.
3. **Random Selection.** Randomly selected genes were used as controls. For each STRING cut-off score, we counted the number of genes present in the linked genes dataset, and randomly selected the same number of genes to create a control dataset. Leave-one-out cross-validation was performed on this control dataset. The process of creating a dataset from randomly selected genes was repeated 25 times for each STRING cut-off score.

### Relative Signal-to-Noise Ratio (rSNR)

**Figure 1:**
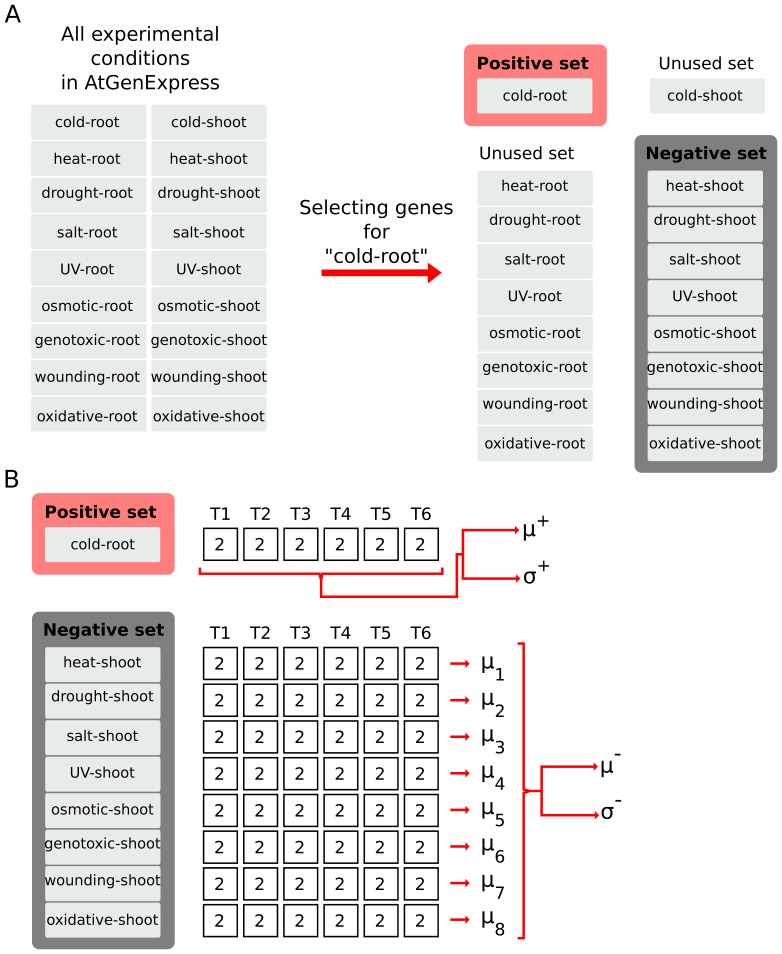
Example showing the calculation of the rSNR for the experimental condition “cold-root” in the AtGenExpress dataset. A) Division of the training dataset into positive and negative sets. The positive set corresponds to the experimental condition of interest (i.e., “cold-root”, in red). Only some of the remaining experimental conditions in the training set, namely those not involving any of the experimental factors that define the experimental condition of interest, are used to build the negative set (in dark gray). B) Calculation of the mean and standard deviation for the positive and negative sets.*T1* to *T6* are time points. Numbers inside the boxes represent the number of replicates for an experimental condition at a given time point. *μ* and *σ* represents the mean and standard deviation respectively. For the positive set, we compute a mean and a standard deviation at each time point. For the negative set, we compute a mean at each time point. We then compute the mean and the standard deviation of the negative set as the mean and the standard deviation of the means computed at each time point respectively.



The rSNR ranks genes according to their association with a given experimental condition. In regards to our classification problem, this means that when the transition profile of a test data is compared with the transition profile of an experimental condition in the training data, only those genes that were found to be relevant for that experimental condition will be used to assess the similarity between the two profiles. In a cross-validation framework, the rSNR gene rank is computed based exclusively on the training data. Hence, the sets of relevant genes are also based exclusively on the training data, and will be different for each cross-validation fold. In order to rank the genes based on their rSNR, for each experimental condition we first define a positive and a negative set in the training data. Basically, the positive set is the training data for the experimental condition of interest, while the negative set comprises the remaining training data not involving any of the experimental factors defining the positive set. For example, let us assume that we are computing the rSNR for the experimental condition involving the experimental factors cold and root (“cold-root”) in the AtGenExpress dataset. In this case, the positive set is the training data for the experimental condition of interest (“cold-root”), while the negative set is the training data involving neither “cold” nor “root”. Other experimental conditions sharing experimental factors with the positive set are excluded from the rSNR calculation. The definition of the positive and the negative set is exemplified in Figure 1A. Next, for all genes we calculate the signal-to-noise ratio in the positive set (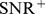) and in the negative set (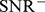) separately. For the experimental condition *k*, the 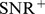 for gene *g* is given by:
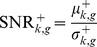
where, 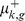 and 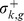 are the mean and the standard deviation respectively of the expression values for gene *g* in the positive set. And the 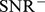 is given by:

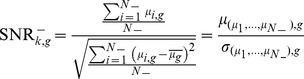
where, 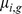 is the mean expression value of gene *g* under experimental condition *i*, 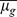 is the mean of the mean expression value of gene *g* across all experimental conditions in the negative set, and 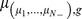 and 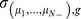 are, respectively, the mean and standard deviation of the expression value of gene *g* across all experimental conditions in the negative set. 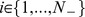 is the set of experimental conditions in the negative set and 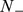 is the total number of experimental conditions in the negative set. Figure 1B illustrates the calculation of the mean and standard deviation for the positive and the negative set.

Finally, we define the rSNR for experimental condition *k* and gene *g* as:
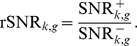



The rSNR can be also be interpreted as the ratio between two coefficients of variation:
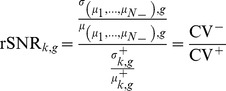
where, 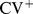 and 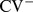 are the coefficients of variation of the positive and negative set respectively. The CV describes the normalized dispersion of the expression level of a gene, i.e., the variability of its expression value with respect to its mean. In microarray experiments, the variability in gene expression levels originates from two sources, technical and biological. Technical variability is primarily controlled using pre-processing, normalization and replication [42]–[45]. Additional technical variability can be assumed to be approximately constant across experimental conditions. In the case of the positive set, most of the biological variability arises from the effect of the experimental factors involved in the experimental condition of interest across time points. Thus, 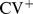 quantifies the biological variability within a particular experimental condition, i.e., “intrinsic noise”. In the case of the negative set, most of the biological variability is due to differences in the effect of the experimental factors considered under the various experimental conditions represented in the dataset. Thus, 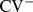 quantifies the biological variability between experimental conditions, i.e., “extrinsic noise”. With regards to the rSNR, this implies that genes that show little variation within the experimental condition of interest but whose expression levels vary across experimental conditions will tend to exhibit high rSNR scores. For the AtGenExpress dataset, we found that genes with high rSNR scores tend to be highly expressed in the positive set, and show little variation across time points, consistent with the expression pattern of fast-response genes to the interventions applied there (data not shown).

The rSNR calculation described above is repeated for all experimental conditions in the training data. Hence, for each experimental condition, we obtain a list of genes with their corresponding rSNR scores, which is then used to select relevant genes. After feature selection, each experimental condition in the training data is represented by a separate list of relevant genes and their corresponding expression values. Subsequently, when test data are compared with a given experimental condition (e.g. “cold-root”), only the genes that were found to be relevant to that particular experimental condition (“cold-root”) are used for similarity score calculation. This process is repeated for each combination of test data and experimental condition in the training data. For a fair comparison with the results based on the link and linked genes datasets, we selected the same number of genes based on their rSNR scores, as obtained for each STRING cut-off score (see previous subsection).

### Comparison with Standard Feature Selection Methods

We compared the performance of the rSNR with two standard feature selection methods. Like the rSNR, these feature selection methods rank genes and select genes based on a ranking:

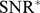
: As previously described, 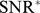 has been extensively used as feature selection method. In particular, it is often used to measure the effectiveness of a feature in discriminating between two classes, and defined as:
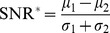
where, *μ_1_* and *μ_2_* are the mean expression value for class 1 and class 2 respectively while *σ_1_* and *σ_2_* are the standard deviation for class 1 and class 2 respectively.
2. **Welch’s t-test:** Welch’s t-test is an adaptation of Student’s t-test to be used on two samples with unequal variance:

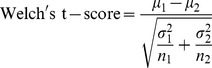
where, *μ_i_*, *σ_i_* and *n_i_* are the mean, standard deviation and sample size, respectively.

It is noteworthy that 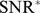 and Welch’s t-test differ only in their estimation of the variance.

### Selecting Optimal Parameters for the Nearest-neighbor Classification

**Figure 2:**
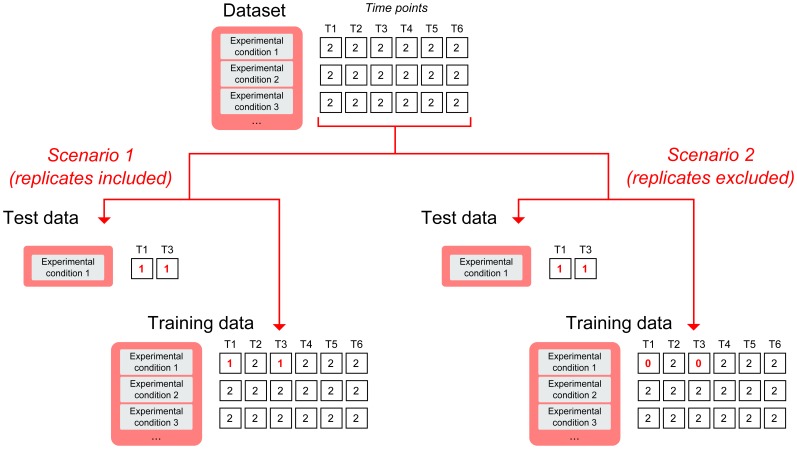
Framework for the evaluation of the performance for methods classifying time-course expression data. In this example, T1 to T6 represent time points. Numbers inside the boxes indicate the number of replicates for each experimental condition at each time point. Test data consists of two time points, T1 and T3, and is taken from the experimental condition 1. In scenario 1, the replicates of the test data are part of the training data, while in scenario 2, the replicates of the test data have been excluded from the training data.

We began by evaluating the performance of our 1NN classifier on the entire AtGenExpress (2074 genes) and EDGE (1600 gene) datasets. These datasets had been previously investigated by Hafemeister et al in the context of time-series microarray classification [30]. Hafemeister et al. suggested to use piecewise constant functions to model time courses, and implemented them as Hidden Markov Models. Parameter estimation and inference was achieved using a Bayesian approach. Since this is a state-of-the-art method, we considered their results as benchmark for comparison with our preliminary analysis. We also used their results as a baseline to improve with our feature selection approach. Since we modified these datasets from the original ones (4 experimental conditions were removed from EDGE dataset, see subsection on datasets), here we report the accuracy obtained by running the Python package available from Hafemeister et al. [30] on the modified datasets. We were also able to reproduce the results reported by Hafemeister et al. on the original dataset (data not shown). Additionally, to make our results comparable, we considered two different scenarios: 1) including replicates of the test data in the training data (as presented by Hafemeister et al.), and 2) excluding replicates of the test data from the training data (see Figure 2). The test data comprised at least 2 time points in scenario 1, and exactly 2 time points in scenario 2. In scenario 1, the test data were defined by randomly selecting 1) the number of time points, 2) the particular time points, and 3) the replicates used. The rest of the data were used as training data. The selected time points were sorted in ascending order according to time, and pairs of consecutive time points were used to generate transition profiles. Then, we compared each transition profile against all possible transition profiles of the training data, and computed the similarity value (Pearson correlation coefficient) between them. Each transition profile in the test data was labeled with the experimental condition for which we obtained the highest similarity value. Finally, the test data were labeled with an experimental condition using majority voting, or highest sum of similarity scores in case of tie. Under scenario 2, replicates of the test data were excluded from the training data; otherwise, we followed the same setup. Note that, if the number of time points selected for the test data equaled the total number of time points available for a particular experimental condition, including one replicate for each time point in the test data and excluding all other replicates from the training data would result in an empty training data for that experimental condition (see Figure S1). To avoid such a situation, under scenario 2 we limited the size of the test data to only 2 time points.

## Results and Discussion

We set out to improve the classification of microarray time series by various means. We evaluated how gene expression profiles from two different time points can be combined, yielding transition profiles. We incorporated protein interaction information into the definition of such transition profiles, and selected genes based on the same information. Finally, we compared different feature selection approaches, and developed the rSNR (relative Signal-to-Noise Ratio) method to extract the genes that are the most relevant to a specific biological condition.

### The Mean Transition Profile (MTP) Performed Best among our Profile Generation Methods

**Figure 3:**
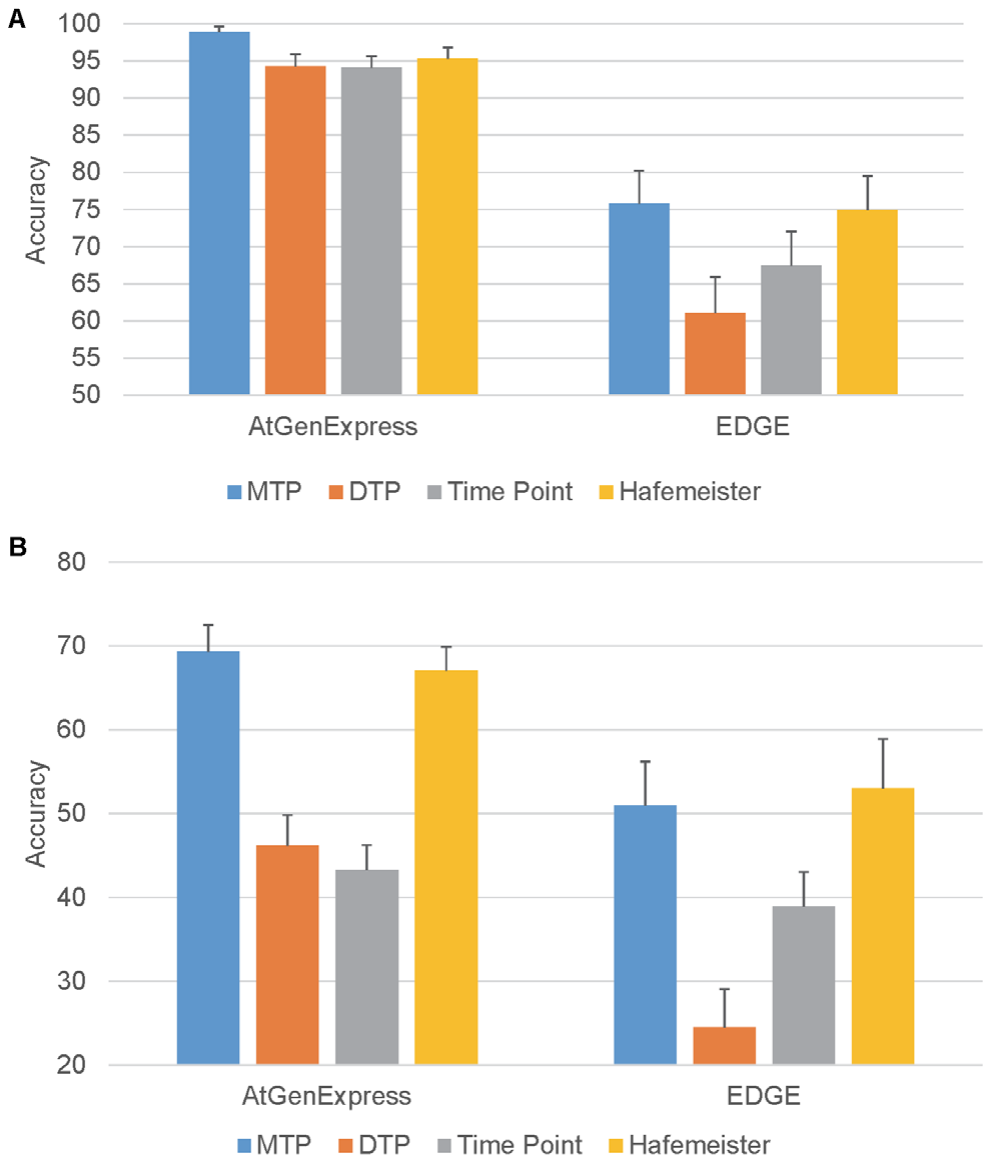
Accuracy of cross-validations using different parameters. A) Accuracy computed under scenario 1 (see Methods). B) Accuracy computed under scenario 2 (see Methods). Error bars indicate 1 standard deviation away from the mean.

We evaluated our method on the AtGenExpress and the EDGE datasets using leave-one-out cross-validation. First, we applied our method to the entire datasets. We compared the performance of the method using different gene expression and transition profiles, as well as with previous work. Figure 3A represents scenario 1, in which replicates of the test data are included in the training data (for better comparability with the results by Hafemeister et al), while Figure 3B represents scenario 2, in which replicates of the test data are excluded from the training data. Among the three expression and transition profiles, MTP performed best in most cases. This suggests that the mean expression value of the genes, rather than their change in expression, is specific to the experimental condition. Moreover, MTP performed better than single time point expression profiles, indicating, as expected, that two time points considered together contain more information than two single time points considered separately for the classification task. Also, MTP performs comparably to the method by Hafemeister et al. Hence, MTP was chosen as the transition profile formula for further evaluations. In addition, all further evaluations exclude the replicates from the training data.

### The rSNR Performed Best among All Evaluated Feature Selection Methods

**Figure 4:**
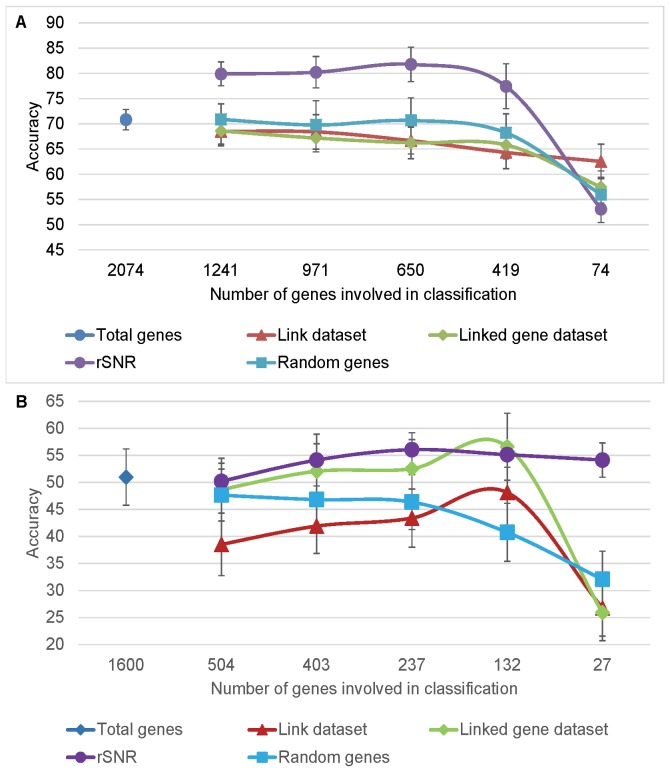
Effect of feature selection methods on the classification of time-course expression data. Accuracy was calculated based on the A) AtGenExpress and B) EDGE datasets. Error bars indicate 1 standard deviation away from the mean.

Next, we evaluated different feature selection methods with the aim of improving classification performance. We compared the performance of gene and link-based methods, using randomly selected genes as controls (see Methods). To render all feature selection methods comparable, the classification decision was always based on the same number of genes, independently of the feature selection method. The results are shown in Figures 4A and 4B. The accuracy of the classifier based on randomly selected genes decreased with the amount of information available for the classification. The decrease in accuracy is gradual, but drops significantly at the end for both AtGenExpress and EDGE datasets, suggesting the existence of a necessary and sufficient number of genes to describe experimental conditions. Both STRING-based feature selection methods failed to perform better than the random selection for AtGenExpress. Only the method based on the linked genes dataset performed better than random for EDGE. Additionally, we observed that the performance of the classifier based on the link dataset was generally not better than that of the linked genes dataset. There are some plausible explanations for the poor performance of STRING-based feature selection methods, and more specifically, the link-based feature selection method:

- The STRING database is not a process-specific protein interaction database. A process-specific database, e.g., a database of interactions involved in stress response of *Arabidopsis*, would probably help in selecting interactions specific to each experimental condition in AtGenExpress.
- Some genes that might be relevant for the experimental conditions under study are not present in STRING, and, hence, information provided by these genes is lost.
- It is disputable whether genes with more links in STRING are more important, or simply more extensively studied.

The rSNR feature selection method exhibited the best overall performance, achieving higher accuracy values compared to randomly selected genes for both datasets. Additionally, applying the rSNR feature selection method generally resulted in a significantly higher accuracy as compared to the other feature selection methods. A detailed analysis of the rSNR method based on the AtGenExpress dataset is presented in the following section.

### rSNR Performed Distinctly Better than Random Gene Selection

**Figure 5:**
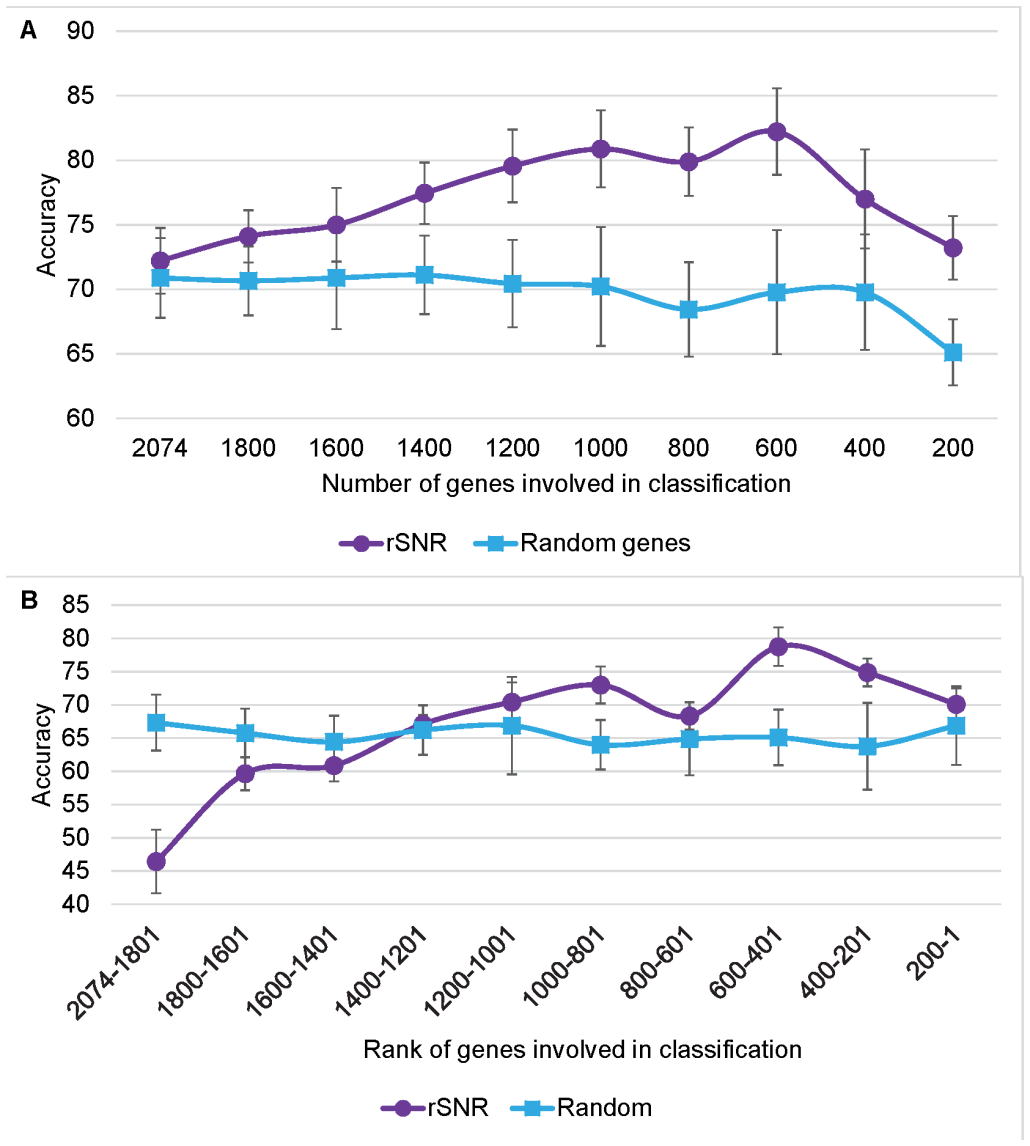
Evaluation of the effect of the rSNR-based rank on the classification of experiments from the AtGenExpress dataset. A) Accuracy was calculated based on gene sets of uniformly decreasing size, selected based on rSNR ranking. B) Accuracy was calculated based on gene sets belonging to different rSNR-based rank sections. Error bars indicate 1 standard deviation away from the mean.

To better understand the rSNR method, we systematically decreased the number of features in the dataset by uniformly removing 200 genes in a step-wise fashion, and compared the performance of the rSNR with control datasets containing the same number of randomly selected genes (Figure 5A). The rSNR performed significantly better than the controls. However, as observed for different STRING cut-off scores (Figure 4A), with a small number of genes, the accuracy obtained using the rSNR becomes similar to random, suggesting, as expected, that there is a minimum number of genes required to distinguish between experimental conditions.

### Relevant Genes Scored High on the rSNR-based Gene Rank

For each experimental condition in the training dataset, we created a gene list, sorted in ascending order (from bottom to top) according to their rSNR scores. For the rSNR to constitute a reliable feature selection method in the context of microarray classification, the genes on the top of the list should be relevant to the specific experimental condition of interest. On the other hand, the bottom of the list should contain genes considered to be noise. To verify that this is indeed the case, we selected 200 genes from different sections of the rSNR-based sorted gene list, and performed cross-validation, reporting average accuracy values. As control, we used 200 randomly selected genes. As shown in Figure 5B, the accuracy of the classifier is minimum when 200 genes are selected from the bottom of the rSNR-based sorted gene list. Accuracy increases gradually as we select genes from higher in the list, until it reaches a plateau. The graph shows that genes at the top of the rSNR-based sorted gene list are more important for the classification than genes at the bottom, suggesting that the rSNR indeed identifies relevant genes for the experimental condition under study.

### Comparing the rSNR with Similar Feature Selection Methods

**Figure 6:**
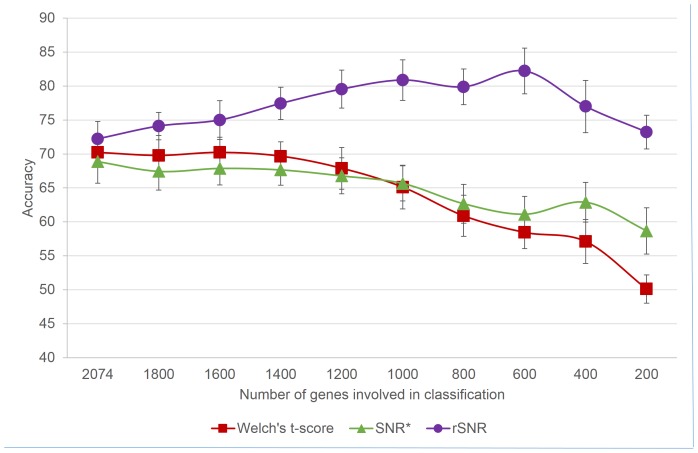
Cross-validation results of the comparison between rSNR and other feature selection methods on the AtGenExpress dataset. Error bars indicate 1 standard deviation away from the mean.

We compared the performance of the rSNR with 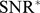 and Welch’s t-test. As shown in Figure 6 and Text S1, the rSNR performs significantly better than the other two feature selection methods.

### Testing Methods on Non-time Series Microarray Data

**Figure 7:**
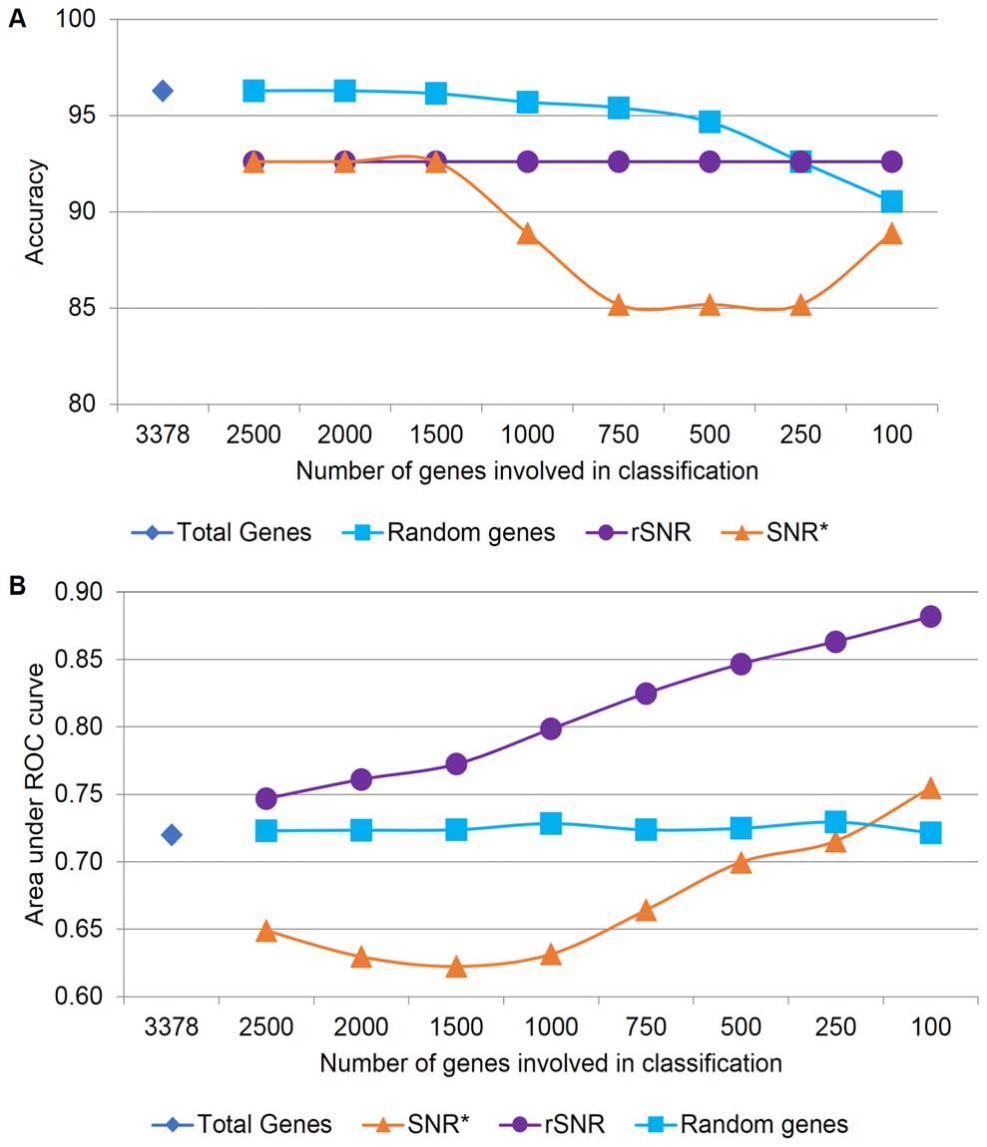
Effect of feature selection methods on the classification of the Engreitz dataset. A) Accuracy. B) Area under the ROC curve (AUC).

In addition to testing our methods on time-series datasets, we applied them to the static microarray dataset from Engreitz et al. Engreitz et al. classified microarray experiments into three disease types, achieving an area under the ROC curve (AUC) of 0.729. As discussed in the Methods section, the application of our method required the modification of the original dataset. Hence, direct comparison with the results of Engreitz et al. is not appropriate. Even randomly selected genes perform extremely well at classifying these data (Figure 7A), an observation reminiscent of results reported by Venet et al. [46]. Here, the rSNR results in a slightly lower accuracy than other methods, but shows a superior performance in terms of the AUC (Figure 7B). A higher AUC indicates a better predictive ability of the rSNR. Thus, in a database search setup, where it is not necessarily the best candidate which is of interest, but rather a set of highly scoring candidates, a high rSNR is a useful feature for improving classification results.

## Conclusions

We first showed that the nearest neighbor method performs comparably to the method developed by Hafemeister et al. We found that for the purpose of classification, mean expression profiles describe time-series transitions based on microarray experiments better than differential expression profiles and single time point expression profiles.

We used biological information as a feature selection criterion wherein a selected feature is known to involve a protein interaction. The source of this biological information was protein interaction information from the STRING database. We investigated the performance of different methods for reducing the high dimensionality of microarray data based on such information. We found that, compared to simple expression profiles, the addition of information on interactions does not provide a clear advantage in the classification of microarray experiments. Moreover, the application of feature selection methods relying on interactions resulted in performances comparable to those obtained with randomly selected genes. Alternative forms of including such information, or the use of interactions from more specific databases, describing the process of interest, might be of advantage.

Finally, we proposed a novel method for feature selection that we called the relative Signal to Noise Ratio (rSNR). The rSNR gives a score to each gene based on its relevance for each experimental condition. This score can then be used to select relevant genes. We showed that the performance of classifiers based on genes with low rSNR scores is substantially worse than that of classifiers based on genes with high rSNR scores. This result indicates that the genes relevant for classification of the experimental condition of interest rank high, in contrast to irrelevant genes, which may be considered noise. Due to its simplicity, our method is particularly attractive for database searches. In a preprocessing step, the microarray datasets for different experimental conditions in the database can be summarized using only the most relevant genes. Hence, when a query microarray is searched against the entire database, instead of comparing the expression profiles of all genes, only the expression profiles of genes relevant to an experimental condition need to be compared. Both in terms of memory and performance, such a procedure would demand relatively few computational resources.

## Supporting Information

Figure S1 **Example of cross-validation when all time points of an experimental condition are selected as test data.**
(TIF)

Text S1 **Results of comparison between rSNR and Significance Analysis of Microarray (SAM).**
(PDF)

## References

[1] Bar-Joseph Z (2004) Analyzing time series gene expression data. Bioinformatics 20: 2493–2503 Available: 10.1093/bioinformatics/bth283. 15130923

[2] Edgar R, Domrachev M, Lash AE (2002) Gene Expression Omnibus: NCBI gene expression and hybridization array data repository. Nucleic Acids Res 30: 207–210. 11752295 10.1093/nar/30.1.207 PMC99122

[3] Guyon I, Elisseeff A (2003) An introduction to variable and feature selection. The Journal of Machine Learning Research 3: 1157–1182 Available: http://dl.acm.org/citation.cfm?id=944968.

[4] Goecks J, Nekrutenko A, Taylor J (2010) Galaxy Team (2010) Galaxy: a comprehensive approach for supporting accessible, reproducible, and transparent computational research in the life sciences. Genome Biol 11: R86 Available: 10.1186/gb-2010-11-8-r86. 20738864 PMC2945788

[5] Kallio MA, Tuimala JT, Hupponen T, Klemelä P, Gentile M, et al (2011) Chipster: user-friendly analysis software for microarray and other high-throughput data. BMC Genomics 12: 507 Available: 10.1186/1471-2164-12-507. 21999641 PMC3215701

[6] Gentleman RC, Carey VJ, Bates DM, Bolstad B, Dettling M, et al (2004) Bioconductor: open software development for computational biology and bioinformatics. Genome Biol 5: R80 Available: 10.1186/gb-2004-5-10-r80. 15461798 PMC545600

[7] Duval B, Hao J-K (2010) Advances in metaheuristics for gene selection and classification of microarray data. Brief Bioinform 11: 127–141 Available: 10.1093/bib/bbp035. 19789265

[8] Beyer A, Bandyopadhyay S, Ideker T (2007) Integrating physical and genetic maps: from genomes to interaction networks. Nat Rev Genet 8: 699–710 Available: 10.1038/nrg2144. 17703239 PMC2811081

[9] Fuente A de la (2010) From ‘differential expression’ to ‘differential networking’ - identification of dysfunctional regulatory networks in diseases. Trends Genet 26: 326–333 Available: 10.1016/j.tig.2010.05.001. 20570387

[10] Chuang H-Y, Lee E, Liu Y-T, Lee D, Ideker T (2007) Network-based classification of breast cancer metastasis. Mol Syst Biol 3: 140 Available: 10.1038/msb4100180. 17940530 PMC2063581

[11] Parsons DW, Jones S, Zhang X, Lin JC-H, Leary RJ, et al (2008) An integrated genomic analysis of human glioblastoma multiforme. Science 321: 1807–1812 Available: 10.1126/science.1164382. 18772396 PMC2820389

[12] Rapaport F, Zinovyev A, Dutreix M, Barillot E, Vert J-P (2007) Classification of microarray data using gene networks. BMC Bioinformatics 8: 35 Available: 10.1186/1471-2105-8-35. 17270037 PMC1797191

[13] Ma X, Lee H, Wang L, Sun F (2007) CGI: a new approach for prioritizing genes by combining gene expression and protein-protein interaction data. Bioinformatics 23: 215–221 Available: 10.1093/bioinformatics/btl569. 17098772

[14] Wu C, Zhu J, Zhang X (2012) Integrating gene expression and protein-protein interaction network to prioritize cancer-associated genes. BMC Bioinformatics 13: 182 Available: 10.1186/1471-2105-13-182. 22838965 PMC3464615

[15] Chopra P, Lee J, Kang J, Lee S (2010) Improving cancer classification accuracy using gene pairs. PLoS One 5: e14305 Available: 10.1371/journal.pone.0014305. 21200431 PMC3006158

[16] Scheubert L, Schmidt R, Repsilber D, Luštrek M, Fuellen G (2011) Learning biomarkers of pluripotent stem cells in mouse. DNA research 18: 233–251 Available: http://dnaresearch.oxfordjournals.org/content/18/4/233.short. 21791477 10.1093/dnares/dsr016 PMC3158465

[17] Staiger C, Cadot S, Kooter R, Dittrich M, Müller T, et al (2012) A critical evaluation of network and pathway-based classifiers for outcome prediction in breast cancer. PLoS One 7: e34796 Available: 10.1371/journal.pone.0034796. 22558100 PMC3338754

[18] Constantinides CD, Atalar E, McVeigh ER (2005) Signal-to-noise measurements in magnitude images from NMR phased arrays. Magnetic Resonance in Medicine 38: 852–857 Available: http://onlinelibrary.wiley.com/doi/10.1002/mrm.1910380524/abstract. 10.1002/mrm.1910380524 PMC2570034 9358462

[19] Daly TM, Dumaual CM, Dotson CA, Farmen MW, Kadam SK, et al (2005) Precision profiling and components of variability analysis for Affymetrix microarray assays run in a clinical context. J Mol Diagn 7: 404–412 Available: 10.1016/S1525-1578(10)60570-3. 16049313 PMC1867543

[20] Mutch DM, Berger A, Mansourian R, Rytz A, Roberts M-A (2002) The limit fold change model: a practical approach for selecting differentially expressed genes from microarray data. BMC Bioinformatics 3: 17. 12095422 10.1186/1471-2105-3-17 PMC117238

[21] Raman T, O’Connor TP, Hackett NR, Wang W, Harvey B-G, et al (2009) Quality control in microarray assessment of gene expression in human airway epithelium. BMC Genomics 10: 493 Available: 10.1186/1471-2164-10-493. 19852842 PMC2774870

[22] Hengpraprohm S, Chongstitvatana P (2009) Feature selection by weighted-SNR for cancer microarray data classification. International Journal of Innovative Computing, Information and Control 5.

[23] Huang CJ, Liao WC (2003) A comparative study of feature selection methods for probabilistic neural networks in cancer classification. Tools with Artificial Intelligence, 2003. Proceedings. 15th IEEE International Conference on. IEEE. 451–458. Available: http://ieeexplore.ieee.org/xpls/abs_all.jsp?arnumber=1250224.

[24] Pomeroy SL, Tamayo P, Gaasenbeek M, Sturla LM, Angelo M, et al (2002) Prediction of central nervous system embryonal tumour outcome based on gene expression. Nature 415: 436–442 Available: http://www.nature.com/nature/journal/v415/n6870/abs/415436a.html. 11807556 10.1038/415436a

[25] Ryu J, Cho SB (2002) Gene expression classification using optimal feature/classifier ensemble with negative correlation. Neural Networks, 2002. IJCNN’02. Proceedings of the 2002 International Joint Conference on. IEEE, Vol. 1: 198–203 Available: http://ieeexplore.ieee.org/xpls/abs_all.jsp?arnumber=1005469.

[26] Slonim DK, Tamayo P, Mesirov JP, Golub TR, Lander ES (2000) Class prediction and discovery using gene expression data. Proceedings of the fourth annual international conference on Computational molecular biology. ACM. pp. 263–272. Available: http://dl.acm.org/citation.cfm?id=332564.

[27] Lakshmi K, Mukherjee S (2006) An improved feature selection using maximized signal to noise ratio technique for TC. Information Technology: New Generations, 2006. ITNG 2006. Third International Conference on. IEEE. pp. 541–546. Available: http://ieeexplore.ieee.org/xpls/abs_all.jsp?arnumber=1611649.

[28] Mishra D, Sahu B (2011) Feature Selection for Cancer Classification: A Signal-to-noise Ratio Approach. International Journal of Scientific & Engineering Research 2.

[29] Goh L, Song Q, Kasabov N (2004) A novel feature selection method to improve classification of gene expression data. Proceedings of the second conference on Asia-Pacific bioinformatics-Volume 29. Australian Computer Society, Inc. pp. 161–166. Available: http://dl.acm.org/citation.cfm?id=976542.

[30] Hafemeister C, Costa IG, Schönhuth A, Schliep A (2011) Classifying short gene expression time-courses with Bayesian estimation of piecewise constant functions. Bioinformatics 27: 946–952 Available: 10.1093/bioinformatics/btr037. 21266444

[31] Kilian J, Whitehead D, Horak J, Wanke D, Weinl S, et al (2007) The AtGenExpress global stress expression data set: protocols, evaluation and model data analysis of UV-B light, drought and cold stress responses. The Plant Journal 50: 347–363 Available: http://onlinelibrary.wiley.com/doi/10.1111/j.1365-313X.2007.03052.x/full. 17376166 10.1111/j.1365-313X.2007.03052.x

[32] Hayes KR, Vollrath AL, Zastrow GM, McMillan BJ, Craven M, et al (2005) EDGE: a centralized resource for the comparison, analysis, and distribution of toxicogenomic information. Molecular pharmacology 67: 1360–1368 Available: http://molpharm.aspetjournals.org/content/67/4/1360.short. 15662043 10.1124/mol.104.009175

[33] Engreitz JM, Morgan AA, Dudley JT, Chen R, Thathoo R, et al (2010) Content-based microarray search using differential expression profiles. BMC Bioinformatics 11: 603 Available: 10.1186/1471-2105-11-603. 21172034 PMC3022631

[34] Jensen LJ, Kuhn M, Stark M, Chaffron S, Creevey C, et al (2009) STRING 8–a global view on proteins and their functional interactions in 630 organisms. Nucleic acids research 37: D412–D416 Available: http://nar.oxfordjournals.org/content/37/suppl_1/D412.short. 18940858 10.1093/nar/gkn760 PMC2686466

[35] Snel B, Lehmann G, Bork P, Huynen MA (2000) STRING: a web-server to retrieve and display the repeatedly occurring neighbourhood of a gene. Nucleic acids research 28: 3442–3444 Available: http://nar.oxfordjournals.org/content/28/18/3442.short. 10982861 10.1093/nar/28.18.3442 PMC110752

[36] Szklarczyk D, Franceschini A, Kuhn M, Simonovic M, Roth A, et al (2011) The STRING database in 2011: functional interaction networks of proteins, globally integrated and scored. Nucleic acids research 39: D561–D568 Available: http://nar.oxfordjournals.org/content/39/suppl_1/D561.short. 21045058 10.1093/nar/gkq973 PMC3013807

[37] Von Mering C, Huynen M, Jaeggi D, Schmidt S, Bork P, et al (2003) STRING: a database of predicted functional associations between proteins. Nucleic acids research 31: 258–261 Available: http://nar.oxfordjournals.org/content/31/1/258.short. 12519996 10.1093/nar/gkg034 PMC165481

[38] Von Mering C, Jensen LJ, Kuhn M, Chaffron S, Doerks T, et al (2007) STRING 7–recent developments in the integration and prediction of protein interactions. Nucleic acids research 35: D358–D362 Available: http://nar.oxfordjournals.org/content/35/suppl_1/D358.short. 17098935 10.1093/nar/gkl825 PMC1669762

[39] Von Mering C, Jensen LJ, Snel B, Hooper SD, Krupp M, et al (2005) STRING: known and predicted protein–protein associations, integrated and transferred across organisms. Nucleic acids research 33: D433–D437 Available: http://nar.oxfordjournals.org/content/33/suppl_1/D433.short. 15608232 10.1093/nar/gki005 PMC539959

[40] Warsow G, Greber B, Falk SSI, Harder C, Siatkowski M, et al (2010) ExprEssence–revealing the essence of differential experimental data in the context of an interaction/regulation net-work. BMC Syst Biol 4: 164 Available: 10.1186/1752-0509-4-164. 21118483 PMC3012047

[41] Ding C, Peng H (2005) Minimum redundancy feature selection from microarray gene expression data. Journal of bioinformatics and computational biology 3: 185–205 Available: http://www.worldscientific.com/doi/abs/10.1142/S0219720005001004. 15852500 10.1142/s0219720005001004

[42] Ritchie ME, Silver J, Oshlack A, Holmes M, Diyagama D, et al (2007) A comparison of background correction methods for two-colour microarrays. Bioinformatics 23: 2700–2707 Available: http://bioinformatics.oxfordjournals.org/content/23/20/2700.short. 17720982 10.1093/bioinformatics/btm412

[43] Smyth GK, Michaud J, Scott HS (2005) Use of within-array replicate spots for assessing differential expression in microarray experiments. Bioinformatics 21: 2067–2075 Available: http://bioinformatics.oxfordjournals.org/content/21/9/2067.short. 15657102 10.1093/bioinformatics/bti270

[44] Smyth GK, Speed T (2003) Normalization of cDNA microarray data. Methods 31: 265–273 Available: http://www.sciencedirect.com/science/article/pii/S1046202303001555. 14597310 10.1016/s1046-2023(03)00155-5

[45] Yang YH, Speed T (2002) Design issues for cDNA microarray experiments. Nat Rev Genet 3: 579–588 Available: 10.1038/nrg863. 12154381

[46] Venet D, Dumont JE, Detours V (2011) Most random gene expression signatures are significantly associated with breast cancer outcome. PLoS Comput Biol 7: e1002240 Available: 10.1371/journal.pcbi.1002240. 22028643 PMC3197658

