# Supplementary figures and images for: Identifying Genes Relevant to Specific Biological Conditions in Time Course Microarray Experiments
Source: PLoS One. 2013 Oct 11;8(10):e76561. doi: 10.1371/journal.pone.0076561 (PMC3795718; doi:10.1371/journal.pone.0076561)

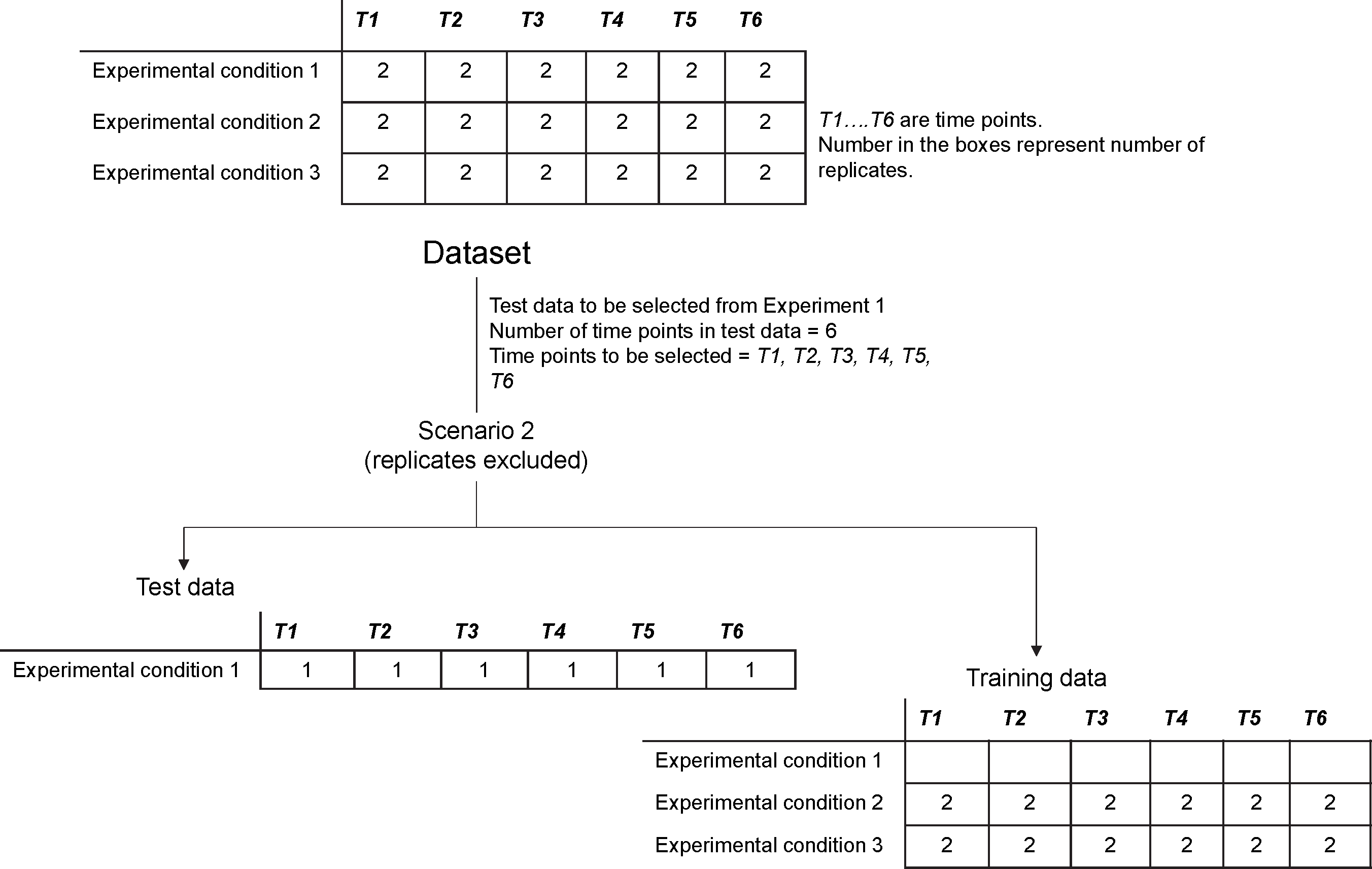

Supplement: Figure S1 — Example of cross-validation when all time points of an experimental condition are selected as test data. (TIF) [file pone.0076561.s001.tif]
