# Supplementary material for: Identifying Genes Relevant to Specific Biological Conditions in Time Course Microarray Experiments
Source: PLoS One. 2013 Oct 11;8(10):e76561. doi: 10.1371/journal.pone.0076561 (PMC3795718; doi:10.1371/journal.pone.0076561)
Supplement: Text S1 — Results of comparison between rSNR and Significance Analysis of Microarray (SAM). (PDF) [file pone.0076561.s002.pdf]

Supporting information for

Identifying genes relevant to specific biological conditions in time course microarray experiments

Nitesh Kumar Singh[1], Dirk Repsilber[2], Volkmar Liebscher[3], Leila Taher[1,*] and Georg Fuellen[1,*]

[1] Institute for Biostatistics and Informatics in Medicine and Ageing Research, Department of Medicine, University of Rostock, Rostock, Germany.

[2] Institute for Genetics and Biometry, Leibniz Institute for Farm Animal Biology, Dummerstorf, Germany.

[3] Institute for Mathematics and Informatics, Ernst Moritz Arndt University of Greifswald, Greifswald, Germany.

* Corresponding authors: Dr. Leila Taher and Prof. Dr. Georg Fuellen, Institute for Biostatistics and Informatics in Medicine and Ageing Research, Ernst-Heydemann-Str. 8, D-18057 Rostock, Germany.

E-mail: leila.taher@uni-rostock.de, fuellen@uni-rostock.de.

Text S1

Comparison with additional feature selection methods

We also compared our method against SAM (Significance Analysis of Microarrays, [1]). SAM can identify genes that are significantly different under different conditions, in different setups. Two of

the available setups are named "two class time course", which uses time series data in two classes as input, and "multiclass", which uses non-time series data in more than two classes as input. Since, our datasets represent multiclass time series experiments, SAM's strategy had to be specifically adapted. We decided to treat our problem as a "two class unpaired time course" problem. For that purpose, as input, we took the positive and negative sets, as defined for the rSNR calculation. SAM's output is a list of significantly "up-regulated" and "down-regulated" genes between the positive and negative set, with scores. This list contained from 100 to 2000 genes, depending on the input. Since we measured the performance of the rSNR for various, fixed numbers of genes, and to make our results comparable, we employed two methods. Assuming we are computing the classification accuracy for N genes:

1. **SAM + Random:** If SAM resulted in n genes, where $n < N$, we randomly sampled N-n genes among the remaining genes, and computed the classification accuracy on the set including both SAM's and randomly sampled genes, for a total of N. if SAM resulted in n genes, where $n > N$, we selected N genes, based on SAM's score.
2. **SAM:** Classification was done exclusively on the genes returned by SAM. For each experimental condition, we counted the number of genes involved in classification. Finally, we computed the average of the number of genes involved in classification.

SAM was implemented using the R package "samr" (http://cran.r-project.org/web/packages/samr/index.html). As shown in the following figure, in our setup, SAM performed systematically worse than the rSNR.

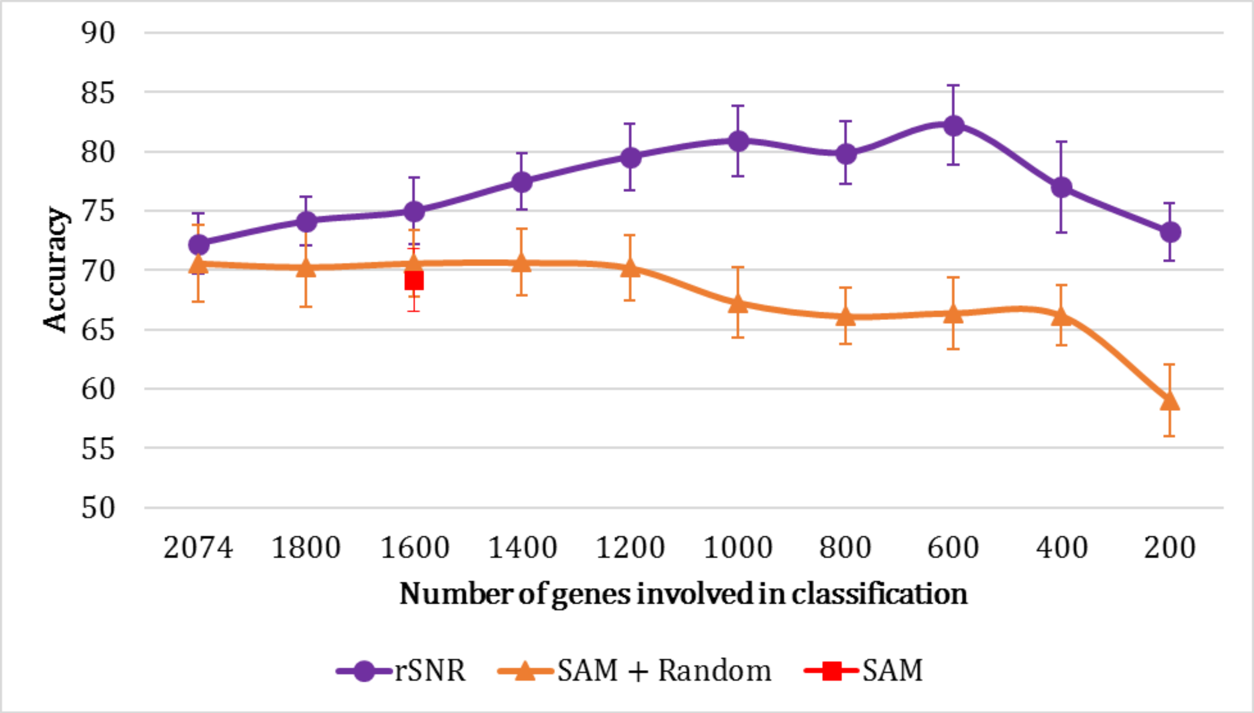

References

1. Tusher VG, Tibshirani R, Chu G (2001) Significance analysis of microarrays applied to the ionizing radiation response. Proc Natl Acad Sci U S A 98: 5116–5121. Available: http://dx.doi.org/10.1073/pnas.091062498.
